# Transvenous ICD Implantation into a Coronary Sinus Branch: A Safe and Feasible Alternative to Deliver ICD after Tricuspid Valve Reconstruction

**DOI:** 10.1155/2023/6646224

**Published:** 2023-02-08

**Authors:** M. Gruszczynski, A. Müller-Burri, A. Häussler, A. Breitenstein, H. Rodriguez Cetina Biefer, O. Dzemali

**Affiliations:** ^1^Department for Cardiac Surgery, Zurich City Hospital Triemli, Zurich, Switzerland; ^2^Department for Cardiology, Zurich City Hospital, Zurich, Switzerland; ^3^Department for Cardiac Surgery, University Hospital Zurich, Zurich, Switzerland; ^4^Department for Cardiology, University Hospital Zurich, Zurich, Switzerland

## Abstract

Significant lead-induced tricuspid regurgitation after cardiovascular implantable electronic devices is not uncommon. Absolute or relative contraindications to place the lead in the right ventricle after tricuspid valve (TV) surgery still remains a challenge. We report about successful lead extraction followed by transvenous implantable cardioverter defibrillator lead placement in the side branches of coronary sinus after TV reconstruction. Furthermore, we discuss therapeutic options to deliver concomitant anti-bradycardia therapy, technical pitfalls, and surgical approaches.

## 1. Introduction

Placement of a transvenous defibrillator (implantable cardioverter defibrillator, ICD) lead in the right ventricular (RV) apex and close to the interventricular septum remains the recommended position for defibrillator coil leads [1].

Significant lead-induced tricuspid regurgitation (TR) after cardiovascular implantable electronic devices (CIED) is not uncommon. Worsening of TR after pacemaker implantation by more than two grades was reported in 18.3% of patients [2].

Absolute or relative contraindications to placing the lead in the right ventricle (RV) after tricuspid valve (TV) surgery remain challenging. In these patients, epicardial patch placement or a subcutaneous ICD (S-ICD) represent possible alternatives. Other non-thoracotomy ICD implantation techniques include placing an ICD lead in the middle branch of the coronary sinus (CS), mid-lateral branch, the cava vein, and the azygous vein [3–6].

We report three successful transvenous ICD lead placements in the side branches of CS after tricuspid reconstruction with a significant improvement of the TV function.

## 2. Cases

### 2.1. Patient No. 1

A 41-year-old female patient underwent a primarily prophylactic dual-chamber ICD implantation due to a dilated cardiomyopathy of unclear etiology in 2008. The left ventricular ejection fraction was initially severely reduced under optimal medical treatment. Five years later, both leads showed dysfunction with raised pacing threshold and presumably insulation defect; therefore, the ICD was extracted entirely and a new system implanted. The device interrogation revealed frequent non-sustained ventricular tachycardia (VT) without needing anti-tachycardia therapy. The patient developed severe TV insufficiency during the consecutive follow-up with symptomatic heart failure The New York Heart Association (NYHA) II–III, elevated N-terminal prohormone of brain natriuretic peptide (NT-proBNP) (483 pg/ml), and repetitive hospital admission. Due to recurrent cardiac decompensation, right-sided heart failure, and profound tricuspid insufficiency, an indication for TV repair was provided. We removed the transvalvular dual coil lead during the operation, closed the cleft between the septal and anterior leaflet, and implanted an annuloplasty ring (Tri-Ad™, 30 mm, Medtronic Schweiz AG, Münchenbuchsee, Switzerland) beating heart through the right-sided mini-thoracotomy. The atrial lead was intentionally abandoned to facilitate the implantation of a new system. Postoperative transesophageal echo only showed residual mild TR. Telemetric monitoring showed an intermittent atrioventricular block and repetitive nonsustained VT with a heart rate of 150 bpm. Subsequently, the patient was discharged to a rehabilitation clinic with a wearable cardioverter defibrillator (WCD, LifeVest™, Zoll Medical, Zoug, Switzerland). It was apparent that the patient would benefit from brady- and anti-tachycardia pacing. The initially evaluated S-ICD lacks these therapeutic options. The decision was made to implant transvenous high voltage (H/V) lead instead of a surgically placed epicardial defibrillator patch and pace/sense lead to avoid repeat thoracotomy. In order to avoid the lead implantation through the repaired TV, we planned the implantation of a transvenous ICD system with a conventional RV single-coil shock lead and a pace–sense electrode placed in the CS venous system. This option has the advantage of being able to deliver anti-tachycardia pacing for VT episodes as well as offering ventricular pacing.

#### 2.1.1. Final ICD Implantation Procedure

**Figure 1:**
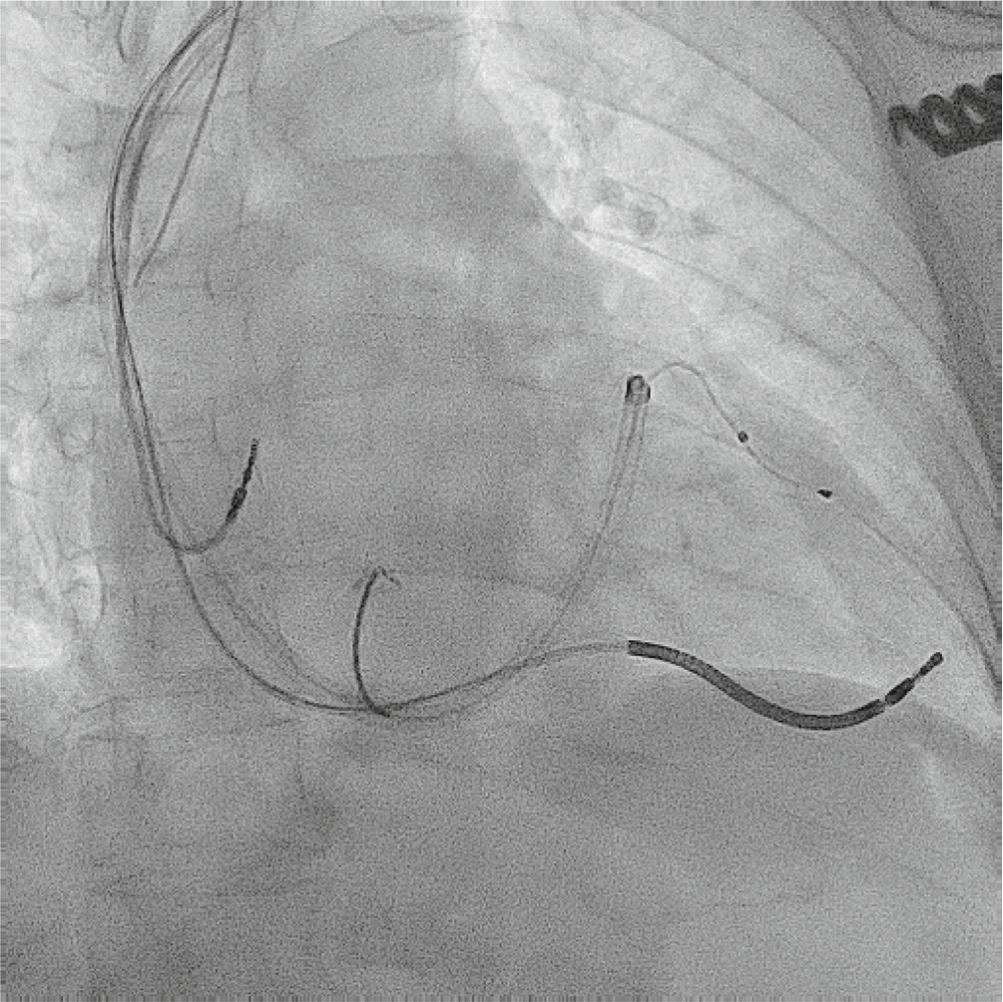
Shock and pace/sense leads in the branches of the coronary sinus.

Under general anesthesia, the ICD system was implanted three months after the tricuspid repair and lead extraction in the electrophysiology (EP) laboratory. The left-arm venography showed evidence of an open left subclavian vein. The skin incision was followed by preparing the deltoid groove and the abandoned atrial lead. The left subclavian venous approach was used to catheterize the CSCS with the 9F/40 cm standard curve Worley Coronary Sinus Guide (CSG) sheath (Merit Medical Schweiz, Zug, Switzerland) and a fixed curved diagnostic EP catheter (Biosense Webster, Johnson & Johnson, Irving, California, USA). The venography showed a large posterolateral vein and a small mid-cardiac vein. The CSG sheath was advanced by telescopic technique in the distal apical portion of the posterolateral vein. Then the 7F single-coil RV shock lead (Durata™ 7122-65, Abbott Schweiz, Baar, Swizterland) was inserted through the CSG sheath in the target vein. The lead interrogation showed sufficient sensing (7 mV), but a high pacing threshold (>10 V at 0.5 ms) and a high impedance of 2000–3000 *Ω*. Therefore, for adequate pacing, a bipolar LV lead (Quickflex™, 1258T-86, Abbott) was implanted in an anterolateral branch of the CS using a second CSG sheath. Measured sensing of this pace–sense lead was >35 mV, with an acceptable pacing threshold of 1.25 V at 0.5 ms and impedance of 1500 *Ω*. The RV lead's DF-1 shock connector and the LV lead's IS-1 pace–sense connector were inserted into the generator's DF-1/RV port and the IS-1 P/S port. The IS-1 connector of the pre-implanted right atrial lead was connected to the RA port, and the needless DF-1/SVC port was plugged (Figure 1). Defibrillation threshold testing was not performed. The measured H/V impedance was 82 *Ω*. The operation time was 97 minutes, and the fluoroscopy was 12 minutes.

#### 2.1.2. Follow-Up

**Figure 2:**
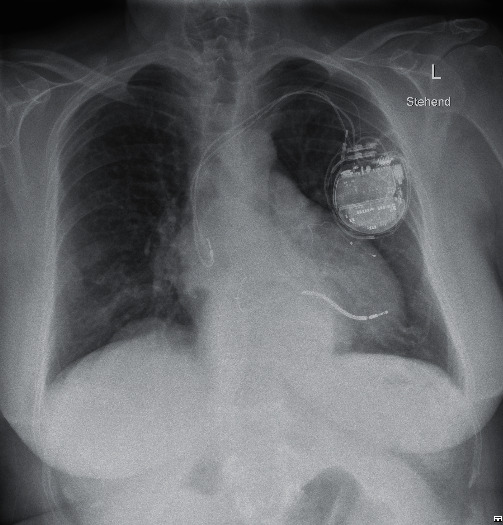
Post-implantation chest X-ray in posterior–anterior view.

**Figure 3:**
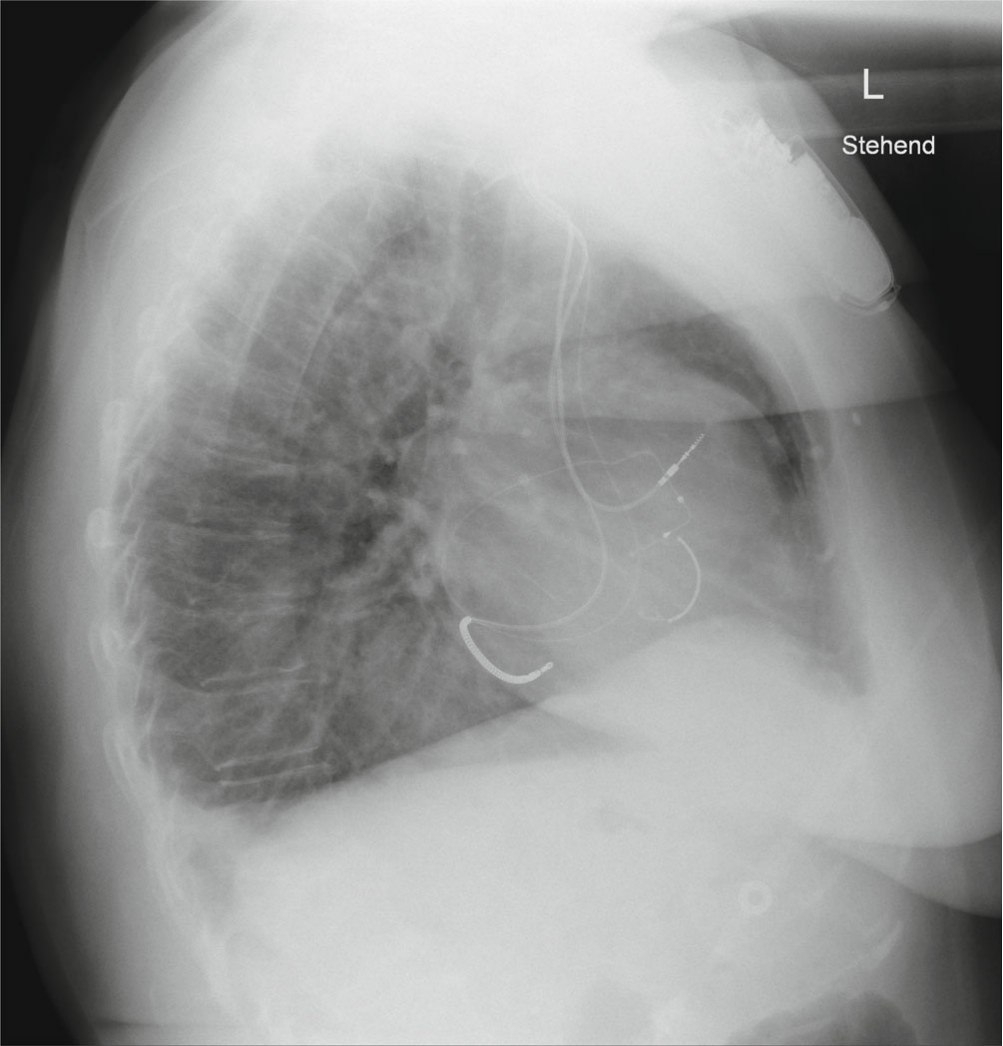
Chest X-ray in lateral view.

The day after the implantation, the chest X-ray showed a small left-sided apical pneumothorax, which showed regression after conservative management. The electrical values were stable (Figures 2 and 3). The patient was discharged home on the second postoperative day. At the post-implant device check, three months postoperatively, there were no late complications, no incidences of lead migration, and no need for re-intervention. The patient presented no symptoms of heart failure, and the TV was competent.

### 2.2. Patient No. 2

A 78-year-old male received a dual-chamber pacemaker due to sick sinus syndrome in 2002. Later, the system was downgraded to one chamber pacemaker, and the atrial lead was abandoned as the patient developed persistent atrial fibrillation. In 2019 with the new onset of symptomatic sustained VT, the one chamber defibrillator was implanted contralateral, and both obsolete pacemaker leads were abandoned in situ. In 2020 the patient developed severe tricuspid insufficiency with symptomatic heart failure NYHA II and elevated NT-proBNP (3076 pg/ml). The left ventricular ejection fraction remained preserved.

Due to the TR caused by both leads, we scheduled the patient for minimal invasive lead extraction and tricuspid repair. The leads could be successfully extracted by a small subclavian incision and excimer laser (GlideLight™, Philips Schweiz, Horgen, Switzerland). We reconstructed the TV with an annuloplasty ring (Tri-Ad™ 34 mm, Medtronic) beating heart via right-sided mini-thoracotomy. Postoperative transesophageal echocardiography showed a competent TV without regurgitation. Subsequently, the patient was discharged to a rehabilitation clinic with a WCD (LifeVest™, Zoll). One month later, we provided the implantation of a new transvenous ICD. To not compromise the repaired TV, the goal was to place the lead inside the CS, thus bypassing the valve.

#### 2.2.1. Final ICD Implantation Procedure

Under general anesthesia, the ICD system was implanted in the EP laboratory. The left-arm venography showed mid-cardiac, small anterior, and anterolateral veins, but no posterior or posterolateral veins.

After probing the mid-cardiac vein with a flexible wire (Sion Blue™ Asahi Intecc, Akatsukichō, Japan), the CSG sheath could be advanced into the mid-cardiac vein with a telescopic technique.

Then we implanted the 7F single-coil H/V lead (Durata™ 7122-65, Abbott) with one IS-1 and DF-1 connector into the apical part of the mid-cardiac vein. Initially, the R wave sensing was 5 mV, pacing threshold >5 V at 0.5 ms, and impedance 1500 *Ω*. Due to the inadequate pacing stimuli, we decided to implant an additional P/S lead in another CS branch.

We finally settled the bipolar S-shaped LV electrode (Quickflex™, Abbott) in a small anterior side branch.

Thus, we achieved sufficient sensing potential of 14.1 mV with a pacing threshold of 0.5 V at 0.5 ms and impedance of 663 *Ω* without phrenic nerve capture.

#### 2.2.2. Follow-Up

Device interrogation revealed ventricular high-rate episodes identified as noise based on the discrimination criteria. These episodes were due to sensing atrial fibrillation through ventricular lead dwelling in the CS near the atrium. In order to avoid inhibition and asystole attributable to this noise and concomitantly detect subtle ventricular fibrillation, we programmed separate sensing for the defibrillation (at 0.3 mV) and stimulation (at 0.7 mV).

One year later, the patient developed severe functional mitral insufficiency with signs of cardiac decompensation, and a mitral clip procedure was performed. The severe insufficiency of mitral valve (MV) was reduced to light-moderate.

### 2.3. Patient No. 3

A 46-year-old female patient suffered from the long QT 7 (Andersen–Tawil syndrome) with preserved left ventricular ejection fraction and received in 2007 as secondary prophylaxis one-chamber dual-coil defibrillator after successful resuscitation due to ventricular fibrillation. In 2014 the lead was explanted in the event of dysfunction, and a new ICD system was implanted ipsilaterally. After that, the patient developed severe TR over the years, which we alleged resulted from the previous lead extraction. The heart team discussed the operation's indication and timing with increasing heart failure symptoms corresponding to NYHA III, elevated NT-proBNP (524 pg/ml), and dilatation of the RV. We decided to schedule the concomitant extraction of the H/V lead with an excimer laser catheter (GlideLight™, Philips) and repair the TV by resuspension of the torn posterior leaflet to the annulus. A bicuspidization of the tricuspid leaflets was performed through an edge-to-edge method.

Additionally, a ring annuloplasty with annuloplasty ring (Tri-Ad™, 32 mm, Medtronic) was performed in the beating heart technique via right-sided mini-thoracotomy. Moreover, the epicardial pacing leads to the atrium and ventricle (CapSure Sense EPI™, Medtronic) were placed and connected with the ICD generator to provide adequate pacing as the patient presented bradycardic sinus rhythm. The postoperative echocardiography revealed a competent TV with a trace of insufficiency, and the patient was bridged with the WCD (LifeVest™, Zoll).

#### 2.3.1. Final ICD Implantation Procedure

One week later, the ICD system was implanted in the EP laboratory under general anesthesia.

Venography showed a small, rapidly branching mid-cardiac vein, and a large anterolateral branch. The first attempt was to advance the LVI catheter and 120° Vein Selector with a Sion Blue wire into the mid-cardiac vein. Due to the atypical exit and the ostial location of the branch, the CSG catheter fell back into the atrium. A renewed probing of the CS enabled advancing the vein selector 2 cm into the distal section of the mid-cardiac vein; therefore, there was not enough backup to push the CSG catheter over the first bifurcation of the mid-cardiac vein. It was impossible to pass the bifurcation with the anchor balloon; therefore, we inserted the shock electrode into the anterolateral branch. The Implantation of the 7F single-coil shock electrode (Durata™, Abbott) over Sion Blue ES wire into the targeted vein was successful.

The DF1 plug was connected to the ICD generator, and the P/S IS1 plug was cut.

We measured a sufficient sensing potential of 5.6 mV with a pacing threshold of 1.5 V at 0.5 ms, shock impedance 64 Ω, and pacing impedance 912 Ω.

#### 2.3.2. Follow-Up

After the rehabilitation, the patient presented no signs of heart failure. The echocardiography showed a good result of the reconstructed TV with average gradients (mean pressure gradient (PG) 3.51 mmHg) and residual slight insufficiency jet. The ICD interrogation revealed the device with regular and stable measured values for sensing atrial (1.1 mV) and ventricular (8.6 mV), pacing threshold (1.25 V/0.4 ms and 0.875 V/0.4 ms, respectively).

The known decreased atrial sensing (0.8 mV) was interpreted without clinical relevance. No ventricular arrhythmias were detected.

## 3. Discussion

Patients with significant TR have significantly reduced prognosis. Moderate-to-severe TR is associated with increased mortality irrespectively of biventricular systolic function and pulmonary pressure. In addition, chronic RV pacing may deteriorate RV function, causing pacing-induced cardiomyopathy, and further annulus dilatation [7]. The reported mean survival from diagnosis of severe TR is 4.35 years, with further reduced survival of 2.28 years after the onset of symptoms [8].

Pacemaker or ICD leads are a potential cause of TV regurgitation. Various mechanisms underlying pacemaker lead-induced TR include direct entrapment, impingement, perforation, and adhesion of the TV leaflets and secondary annulus dilatation.

As transvalvular coil lead placement is contraindicated in patients with a mechanical prosthesis, placing leads through the bioprosthetic or repaired valve has been associated with valve leaflet damage and should be avoided not to cause a new insufficiency.

In the case of lead-induced TR, complete removal of CIED leads is recommended, and there are numerous alternatives to provide other adequate CIED therapy. Some authors describe repositioning pacemaker leads under direct vision and fixation to the annulus of the posterior leaflet during TV repair [9].

Others emphasize avoiding contact between the lead and not only the tricuspid leaflets, but also the tricuspid apparatus. They describe the technical approach of pulling and fixing the lead at the tricuspid annulus to prevent it from floating in the RV. The annuloplasty ring is then attached to the fixed lead to keep the lead from touching the TV [10].

Our priority was to provide permanent pacing and transvenous cardiac defibrillation for patients with previous TV surgery and TR. We prefer the minimally invasive approach to TV repair in the beating heart technique without cardiac ischemia.

The advantages of performing a minimally invasive beating heart TV reconstruction include reduced risk of systemic embolization by avoiding aortic cross-clamp, no myocardial ischemic time, and shorter operation time with comparable results with conventional surgery [11].

The first solution to implement during the mini-thoracotomy is placing either two patch electrodes or one patch electrode and a superior vena cava coil leads; however, at the cost of high lead failure of 20% [12].

Staged implantation of epicardial defibrillator patch electrodes requires a re-thoracotomy and is associated with higher morbidity and mortality. Furthermore, a higher incidence of lead failure of 7.5% in 4 years has been reported [19].

The alternative to transvenous lead placement could be the implantation of subcutaneous coil leads. As an extravascular system, it does not interfere with the TV and is connected with less systemic general infections [20]. The downside is that it does not address the need for pacing therapy; in addition, VT detection threshold does not allow for recognizing slow VT episodes, which can be very important for the patients.

Several transvenous approaches have been described previously, including the use of a floating double-coil in the inferior vena cava [4], placing the ICD lead in the azygous vein [5], the use of a CS defibrillation coil coupled with a left-sided array [13], and implantation of the ICD lead in the low right atrium [14].

A promising and safe technique for implantation of the ICD lead into the middle cardiac vein (MCV) with excellent results from the long-term follow-up have been described [15, 16, 17].

This implantation technique is well known for pacing leads in cardiac resynchronization therapy. However, the target vessel for cardiac resynchronization is the CS's posterolateral or lateral vein. As much as possible, the H/V lead placement should occur in the MCV to include LV muscle mass in the shock vector [18].

Since the anatomy of the CS has high variability, planning, and distribution of the leads in the differentiated CS branches, different connector options should be considered preoperatively.

Although the planned approach for ICD lead was the MCV, the small diameter of the target vessel led us, in one case, to place the coil in the alternative branches. Due to the low pacing values that could not be accepted for a patient with a pacing indication, there was a need to place an additional P/S lead into the anterolateral vein branch or epicardially.

The risks for complications of electrode placement within the CS venous branch-like lead dislodgement, CS perforation, vein thrombosis, or the mortality and morbidity associated with later lead extraction should be critically evaluated.

In conclusion, we report the transvenous placement of a combined P/S-H/V defibrillation lead into the postero-lateral and mid-cardiac vein, respectively, and P/S lead in the anterolateral branch of the CS in the presence of very narrow mid-cardiac vein. The awareness of such necessity and the technical difficulty led us to place upfront epicardial P/S leads in another case. Furthermore, we address the technical issues and possible solutions during the implantation procedure. After restoring tricuspid competence, the heart failure symptoms could have been eliminated. In one case, a newly detected mitral regurgitation was successfully treated with mitral clip, consecutively alleviating the heart failure symptoms.

Our report shows that implantation of ICD lead with or without accessory pace–sense lead into a CS branch is a safe and effective option for delivering brady- and anti-tachycardia therapy in patients with limited trans-tricuspid access to the RV.

## Data Availability

Data supporting this research article are available from the corresponding author or first author on reasonable request.

## Abbreviations

ICD: Implantable cardioverter defibrillator
TR: Tricuspid regurgitation
RV: Right ventricle
CIED: Cardiovascular implantable electronic devices
S-ICD: Subcutaneous ICD
NYHA: The New York Heart Association
WCD: Wearable cardioverter defibrillator
H/V: High voltage
VT: Ventricular tachycardia
EP: Electrophysiology
CS: Coronary sinus.
